# Tumor *Trp53* status and genotype affect the bone marrow microenvironment in acute myeloid leukemia

**DOI:** 10.18632/oncotarget.19042

**Published:** 2017-07-06

**Authors:** Rodrigo Jacamo, R. Eric Davis, Xiaoyang Ling, Sonali Sonnylal, Zhiqiang Wang, Wencai Ma, Min Zhang, Peter Ruvolo, Vivian Ruvolo, Rui-Yu Wang, Teresa McQueen, Scott Lowe, Johannes Zuber, Steven M. Kornblau, Marina Konopleva, Michael Andreeff

**Affiliations:** ^1^ Department of Leukemia, Section of Molecular Hematology and Therapy, The University of Texas MD Anderson Cancer Center, Houston, TX, USA; ^2^ Department of Lymphoma and Myeloma, The University of Texas MD Anderson Cancer Center, Houston, TX, USA; ^3^ Department of Neurosurgery, The University of Texas MD Anderson Cancer Center, Houston, TX, USA; ^4^ Department of Cancer Biology and Genetics, Memorial Sloan Kettering Cancer Center, New York, NY, USA; ^5^ Research Institute of Molecular Pathology, Vienna, Austria

**Keywords:** AML, microenvironment, GEP, stroma, genotype

## Abstract

The genetic heterogeneity of acute myeloid leukemia (AML) and the variable responses of individual patients to therapy suggest that different AML genotypes may influence the bone marrow (BM) microenvironment in different ways. We performed gene expression profiling of bone marrow mesenchymal stromal cells (BM-MSC) isolated from normal C57BL/6 mice or mice inoculated with syngeneic murine leukemia cells carrying different human AML genotypes, developed in mice with *Trp53* wild-type or nullgenetic backgrounds. We identified a set of genes whose expression in BM-MSC was modulated by all four AML genotypes tested. In addition, there were sets of differentially-expressed genes in AML-exposed BM-MSC that were unique to the particular AML genotype or *Trp53* status. Our findings support the hypothesis that leukemia cells alter the transcriptome of surrounding BM stromal cells, in both common and genotype-specific ways. These changes are likely to be advantageous to AML cells, affecting disease progression and response to chemotherapy, and suggest opportunities for stroma-targeting therapy, including those based on AML genotype.

## INTRODUCTION

AML is a heterogeneous hematological disorder characterized by the accumulation of hematopoietic progenitor cells with acquired somatic genetic alterations. Such alterations affect normal mechanisms of self-renewal, proliferation, and differentiation, and give the abnormal hematopoietic cells a growth/survival advantage over their normal counterparts. There is a large body of evidence indicating that the AML cell genotype underlies observed variations in the response of patients to chemotherapy; it is therefore not surprising that leukemia cells can modify their BM microenvironment, and thus “educate it” to promote leukemia progression. We have previously demonstrated that reciprocal interactions between the BM microenvironment and AML cells can modify the BM-MSC transcriptome promoting resistance to chemotherapy *via* processes involving cell-to-cell and cell/matrix interactions, as well as paracrine and autocrine signaling [1]. Similarly, several reports have described critical molecular changes in the stroma of solid tumors [2–4] and more recently; it has been shown in colorectal cancer (CRC) that genes expressed by stromal cells are better predictors of response to therapy and disease prognosis than genes expressed by epithelial tumor cells [4]. In hematological malignancies, BM-MSC from myelodysplastic syndrome (MDS) and AML patients often exhibit altered gene expression profiles, an aberrant phenotype, and abnormal functional properties compared to normal donor-derived BM-MSC [5]. Although many studies support these findings [6–8], little is known about how genetic aberrations in tumor cells differentially impact the genetic and phenotypic changes in the tumor stroma. In this study, we show specific and common transcriptional changes in BM-MSC driven *in vivo* by leukemia cells harboring different genotypes.

## RESULTS

### BM-MSC exposed to leukemia *in vivo* show changes in their gene expression profile

**Figure 1:**
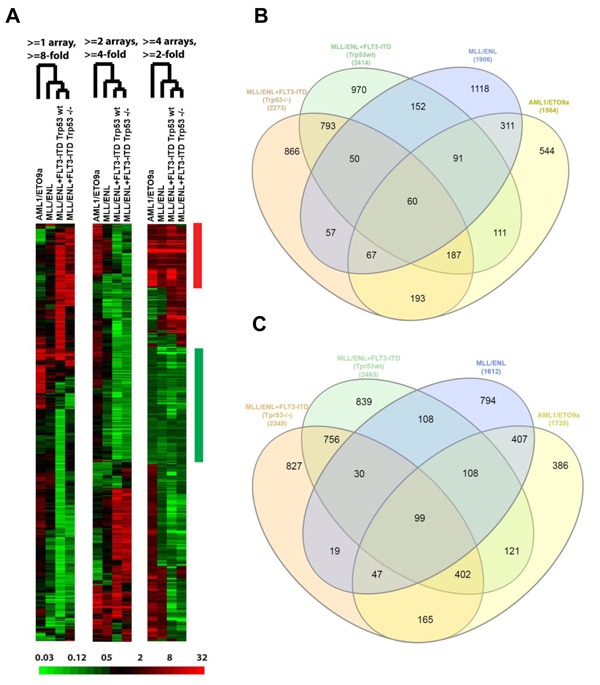
BM-MSC transcriptome regulation by AML **A.**, Three “subtracted” heat maps showing the relative change in expression, according to AML genotype, for genes selected by different criteria. In each heat map, the fold change relative to control BM-MSC is represented according to the color bar at the bottom. Each heat map has been hierarchically clustered for similarity between genes (rows) in the pattern of fold change across compared samples (columns), and for similarity between compared samples (labeled by genotype) in the pattern of gene expression changes. Similarity of genotype effects is indicated by the branched dendrogram, with the two MLL/ENL+FLT3-ITD genotypes (differing by *Trp53* status) giving the most similar changes. Criteria for gene selection at the top indicate the number (1-4) of comparisons (“arrays”) and fold-change magnitude (up or down) required. The bars at the right indicate groups of “common” genes in which the fold-change requirement has been met in the same direction, up (red) or down (green), for all 4 comparisons at the > = 2-fold change level. **B.**-**C.**, Overlap between AML genotypes of genes with altered expression in BM-MSC. Gene probes with a fold-change from control of at least 2 in at least one AML genotype were identified, then numbers of genes were plotted using Venn diagrams according to overlap between genotypes for genes whose expression went up (B) or down (C) in AML-exposed BM-MSCs.

We inoculated C57BL/6 mice with syngeneic primary murine leukemia cells null for *Trp53* (*Trp53* ^-/-^) and harboring one of the following human AML genotypes: AML1/ETO9a, MLL/ENL, or MLL/ENL+FLT3-ITD. To assess the contribution of p53 expression in leukemia cells to changes in their BM-MSC, we also inoculated mice with MLL/ENL+FLT3-ITD primary leukemia cells expressing wild-type (*wt*) *Trp53* [9]. Leukemia engraftment was confirmed by bioluminescence imaging (IVIS) and was allowed to progress for 2 to 4 weeks based on leukemia burden. All types of leukemia were allowed to progress to a similar burden stage based on bioluminescence (data not shown) to adjust for tumor size. For each of the 5 conditions (4 groups corresponding to 4 types of AML, plus 1 group corresponding to control healthy mice), bone marrow was harvested from 5 mice and pooled, an accepted practice for gene expression profiling (GEP) [10]. Duplicate pools (representing a total of 10 mice) were used for each of the AML1/ETO9a and MLL/ENL conditions, and for the control group harvested on the same day. BM-MSC were isolated by fluorescence activated cell sorting (FACS) using the cell surface marker phenotype Ter119-, CD31-, CD45-, CD105+, Sca1+, CD106+, and PDGF-Rα+ (CD140) [11, 12] (Supplementary Figure 1). GEP of RNA from pool-derived sorted BM-MSC samples was performed with Illumina mouse WGv2 BeadArrays. Heat-mapping showed high similarity between duplicate pools (Supplementary Figure 2), supporting data quality and the averaging of data from duplicate pools. After averaging duplicate results and subtracting log2-transformed data for AML-exposed BM-MSC by data for control BM-MSC harvested on the same day, samples for MLL/ENL+FLT3-ITD genotypes (*Trp53 wt* and *Trp53* ^-/-^) were nearest neighbors, and also clustered next to the data for MLL/ENL leukemia cells (Figure 1A). The subtracted data were evaluated with respect to similarity and differences between AML genotypes in their effect on gene expression by BM-MSC. Specifically, gene probes with a fold-change from control (up or down) of at least 2 in at least one genotype were identified (Supplementary Table 1), and their overlap displayed in Venn diagrams (Figure 1B and 1C).

#### Common gene expression changes induced by AML independent of genotype

**Figure 2:**
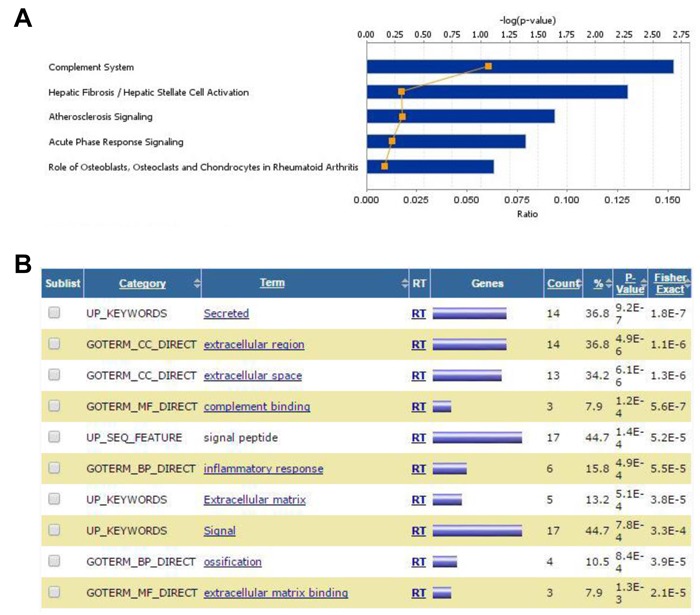
Gene expression analysis of overlapping DEG in BM-MSC from mice engrafted with AML cells **A.**, IPA Stacked Bar Chart of the most significant Canonical Pathways associated with the commonly up- and down-regulated genes induced in BM-MSC by all 4 genotypes. Bars indicate the -log of p-value of the association with the corresponding pathways and reflect the likelihood that the association or overlap between the genes in the set and a given process/pathway is due to random chance. The smaller the p-value the less likely the association is random. The number of molecules in the dataset represented in each network is expressed as ratio (orange line). The significance values (-log of p-value, upper axis) for the canonical pathways is calculated by Fisher's exact test right-tailed. **B.**, DAVID Functional Annotation Chart report for commonly up- and down-regulated genes induced in BM-MSC by all 4 genotypes. The chart shows an annotation-term-focused view of enriched sets (Terms) for the genes in the analysis list based on comparisons with the original databases (Categories). The higher the number (Count) of genes in the list associated with a particular category (expressed as a percentage of the total number of genes in the comparison set (%), the higher the P-value. This P-value is calculated as a modified Fisher Exact P-Value, for gene-enrichment analysis. It ranges from 0 to 1 where P-Value = 0 represents perfect enrichment. Usually, P-Value is equal or smaller than 0.05 to be considered strongly enriched in the annotation categories

GEP analysis revealed sets of genes that were consistently up- or down-regulated in BM-MSC by all four AML genotypes *in vivo* (Figure 1B and 1C and Supplementary Table 1). Notable among the 60 up-regulated genes were *CTGF* (average fold increase [FI] of ~5 by 2 array probes), *IGFBP5* (FI = 3.78 by 3 probes), genes related to complement (*C4A, C4B,* and *SERPING1*), *MGP* (matrix Gla protein, an ECM regulator of multiple types of mesenchymal differentiation, FI = ~3) and *CXCL12* (FI = ~3), which encodes SDF-1α, the master chemo-attractant of normal and malignant hematopoietic precursors. Ingenuity Pathway Analysis (IPA) of commonly up- and down-regulated genes induced by all 4 genotypes indicated activation of pathways related to chronic inflammation (Figure 2A), consistent with the concept that there is a causal link between inflammation and cancer [13–15]. Analysis of upstream regulators using IPA revealed activation of STAT3 and the involvement of other transcription regulators like CTNNB1 (β-catenin) and SMARCA4 (Table 1). Interestingly, SMARCA4 (also known as BRG1) is a transcriptional regulator that can control the activity of Sonic hedgehog and GliA and their downstream targets, providing a driving force for tumor proliferation by activating mitogenic genes such as *GLI1*, *CCND1* and *MYC* [16, 17]. In addition, we analyzed the list of genes that were collectively up-regulated in all 4 conditions using DAVID bioinformatics analysis; a summary of the findings is presented with a functional annotation chart in Figure 2B. Consistent with IPA results, DAVID analysis also indicated an enrichment of genes associated with inflammatory response, complement activation and osteogenic differentiation (Supplementary Table 2). The 99 genes down-regulated in common included *LTF*, a tumor suppressor and inhibitor of AKT signaling [18], and *PDCD4*, a tumor suppressor gene whose decreased expression has been implicated in the development and progression of several types of cancer [19–22].

**Table 1:** IPA Upstream Regulator Analysis for up- and down-regulated genes overlapping in all 4 conditions

Upstream Regulator	Predicted Activation State	Activation z-score	p-value of overlap	Target molecules in dataset
STAT3	Activated	2.207	0.00115	CCNC,CCND2,CXCL12,HP,IGFBP5,LTF,TNF
SMARCA4		1.998	0.00439	CTGF,IGFBP5,INHBA,MGP,PLPP3,PTP4A2,SERPINH1,SPP1
CTNNB1		1.206	0.0028	BGN,CAPNS1,CCND2,CTGF,CXCL12,ECM1,IGFBP5,SPP1
STAT5		1.131	0.0358	CCND2,CISH,TNF,TPM3

Transcriptional regulators identified by IPA as candidates for modulating the gene expression changes observed in our experimental dataset. The analysis predicts which upstream regulators are activated or inhibited to explain the up-regulated (red) and down-regulated (green) genes observed in the dataset. The overlap p-value measures whether there is a statistically significant overlap between the dataset genes and the genes that are regulated by a transcriptional regulator. It is calculated using Fisher's Exact Test, and significance is generally attributed to p-values < 0.01. The calculated z-score reflects the overall predicted activation state of the regulator where z-scores greater than 2 (activation) or smaller than-2 (inhibition) can be considered significant.

### Differential gene expression changes in BM-MSC driven by different AML genotypes

We also compared GEP data for AML-exposed BM-MSC to control BM-MSC separately for each AML genotype. From the analysis of differentially-expressed genes (DEG) as above, and by Gene Set Enrichment Analysis (GSEA), results suggested that AML with different genotypes shaped the transcriptome of BM-MSC in a very specific manner, characterized by distinct cellular functions and canonical pathways.

#### Changes induced by exposure to AML1/ETO9a leukemia

**Figure 3:**
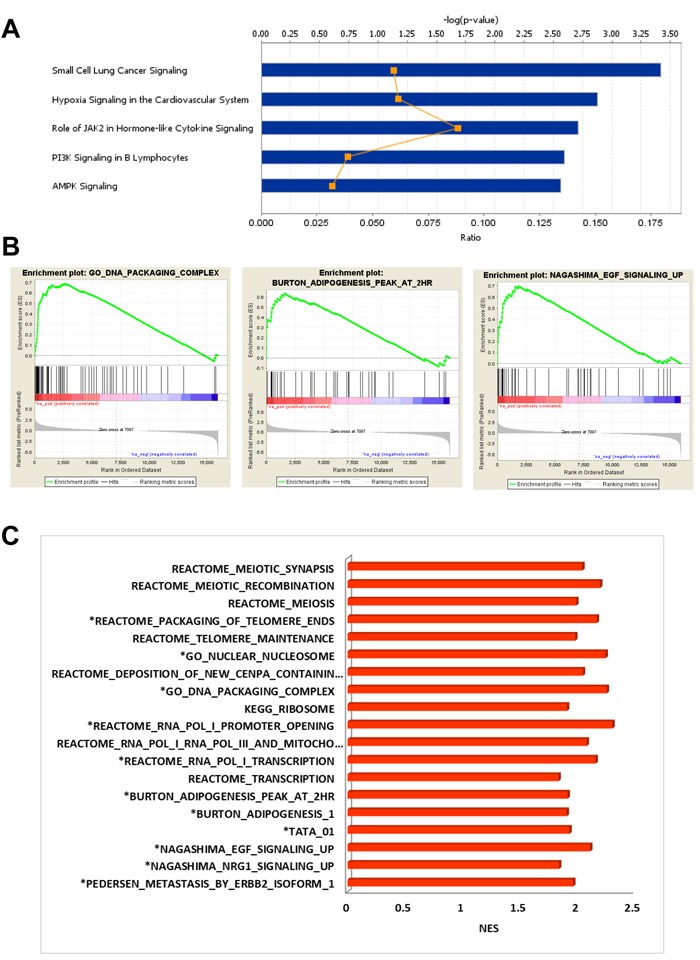
Analysis of DEG in BM-MSC exposed to AML1/ETO9a leukemia **A.**, IPA Stacked Bar Chart of the most significant Canonical Pathways associated with up-regulated genes induced in BM-MSC by AML1/ETO9a leukemia. **B.**, Examples of enrichment plots for the most significant enrichment sets for this dataset. Only enrichment sets with a normalized enrichment score (NES) ≥1.8 and ≤1.8 were considered as positive and negative associated respectively. **C.**, Bar chart summarizing the most significant enrichment sets for this dataset.

In BM-MSC samples isolated from mice bearing the AML1/ETO9a leukemia, GEP analysis indicated that 1564 genes were up-regulated at least 2-fold as compared to the expression in BM-MSC from control mice, 544 of which were not identified in other AML genotypes (Figure 1B). IPA analysis of these unique up-regulated genes indicated that some genes were associated with cell transformation during cancer progression (*ABL1, TRIO, FOSB, NRCAM, TRIB2* and *CDK6*) and others with cell survival (e.g., *BCL10, BBC3, FGF8, HSPA4* and *SOD1*). Furthermore, their functional associations had the lowest p-values (≤ 2.5×10^-4^) suggesting that they are highly significant and unlikely to be random (Supplementary Table 3). The canonical pathways module of IPA revealed that these up-regulated genes participate in pathways related to cancer, hypoxia, AMPK, PI3K and JAK2 signaling (Figure 3A). Analysis of upstream regulators suggested activation of FOXO3 and EPAS1 (HIF2A) and inactivation of KDM5A, a transcription regulator with histone demethylase functions. IPA of down-regulated genes in BM-MSC exposed to AML1/ETO9a leukemia *in vivo* suggested a decrease in immune response functions (activation z-score -2.200)

GSEA for BM-MSC exposed to AML1/ETO9a leukemia, as compared to control BM-MSC, found significant enrichment (FDR < 5%) for a short list of gene sets clearly different from those obtained for other genotypes and associated with meiosis, mitochondrial transcription, telomere maintenance and ribosome components. The up-regulation of genes coding for histones and histone family members correlated with a positive enrichment for gene sets associated with chromosome organization, DNA packaging, chromatin organization and chromatin silencing (Supplementary Table 4), although some of these sets were not unique to this condition. Interestingly, there was overlap with gene sets involved in adipogenesis and EGF signaling (Figure 3B-3C). Consistent with IPA findings, negatively-enriched gene sets included ones with positive regulation of IL-6 and IL-8 production and positive regulation of inflammatory response, suggesting a decrease in the production of cytokines after exposure to AML1/ETO9a and a reduced inflammatory response.

#### Changes induced by exposure to MLL/ENL leukemia

**Figure 4:**
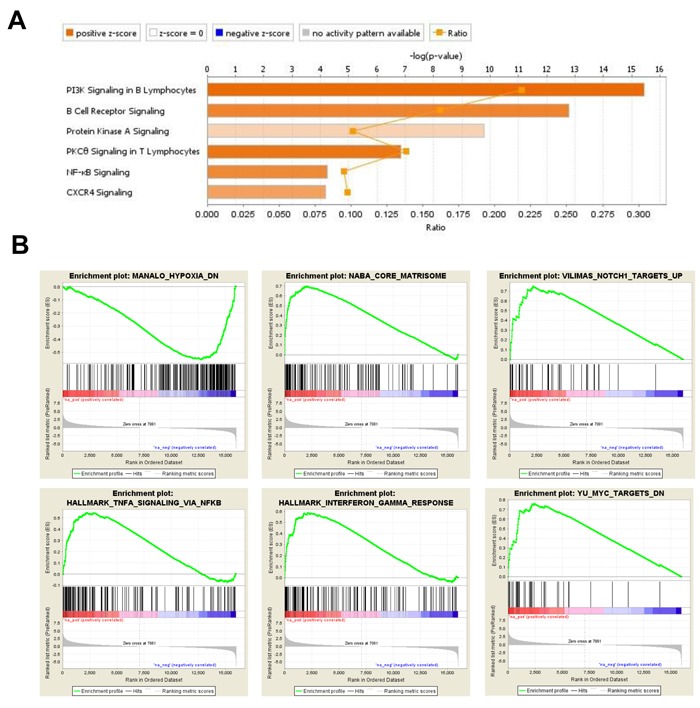
DEG in BM-MSC engrafted with MLL/ENL **A.**, IPA displaying the most significant Canonical Pathways associated with up-regulated genes induced by MLL/ENL leukemia in BM-MSC. The Stacked Bar Chart displays the up-regulated genes molecules in each Canonical Pathway with regards to their z-score. The color orange reflects a positive direction of change for the function. Z-scores ≥ 2 indicate that the function has a statistically significant increase. **B.**, Examples of enrichment plots for the most significant enrichment sets for this dataset. Only enrichment sets with a normalized enrichment score (NES) ≥1.8 and ≤1.8 were considered as positive and negative associated respectively.

BM-MSC samples isolated from mice bearing MLL/ENL leukemia, without FLT3 abnormality, were found to have a total of 1906 genes up-regulated and 1612 down-regulated, by at least 2-fold as compared to control BM-MSC (Figure 1B and 1C). IPA analysis of the 1118 uniquely up-regulated genes suggested activation of PI3K, PKCθ, PKA, phospholipase C (PLC), NF-κB and CXCR4 signaling, among other canonical pathways (Figure 4A and Supplementary Table 5). Cellular functions like cell movement, migration, invasion, infiltration and chemotaxis were highly represented and had significantly high activation scores. Among the most significant upstream regulators of these genes, we found RELA (p65 subunit of NF-κB), FOXO1, HTT, SMARCA4, CTNNB1, STAT1, STAT3 and HIF1A (Supplementary Table 6). Consistent with these findings, GSEA positively enriched gene sets associated with hypoxia, stromal stimulation, extracellular matrix (ECM) and positive regulation by NOTCH1, TGF-β, INFγ and TNF-α *via* NF-κB (Figure 4B). For the 794 uniquely down-regulated genes, IPA indicated activation of aryl hydrocarbon receptor (AHR) signaling and a decrease in NRF2-mediated oxidative stress response. Analysis of upstream regulators pointed at MYC, HIP2K and NRF2 transcription factors as being inhibited or not activated under these conditions. Interestingly, it has been shown in a mouse model of lung cancer that NRF2-deficiency in mice creates an immunosuppressive microenvironment with increased metastatic potential [23]. GSEA also indicated MYC inhibition.

#### Changes induced by exposure to MLL/ENL+FLT3-ITD leukemia

For BM-MSC exposed to both forms (*Trp53* wt and *Trp53*^-/-^) of MLL/ENL+FLT3-ITD genotypes, 793 up-regulated genes and 756 down-regulated genes were exclusively shared between the two conditions and differentially expressed at least 2-fold as compared to control BM-MSC (Figure 1B and 1C). Genes coding for ECM proteins like *BMP1,* *SPON2* and *TGM2* (Transglutaminase 2) were notably up-regulated. Interestingly, overexpression of TGM2 has been linked to resistance to chemotherapy [24, 25]. For both *Trp53* wt and *Trp53*^-/-^ forms of MLL/ENL+FLT3-ITD, GSEA indicated positive enrichment for gene sets associated with activation of β-catenin and with genes up-regulated in hematopoietic stem cells (HSC) compared to other cell types, among others. These findings suggest that these transcriptome changes could be driven by the presence of FLT3-ITD in the leukemia compartment, as in general, they were substantially different from the ones observed in BM-MSC exposed to MLL/ENL leukemia.

### *Trp53* status in leukemia cells impacts the BM microenvironment transcriptional response

**Figure 5:**
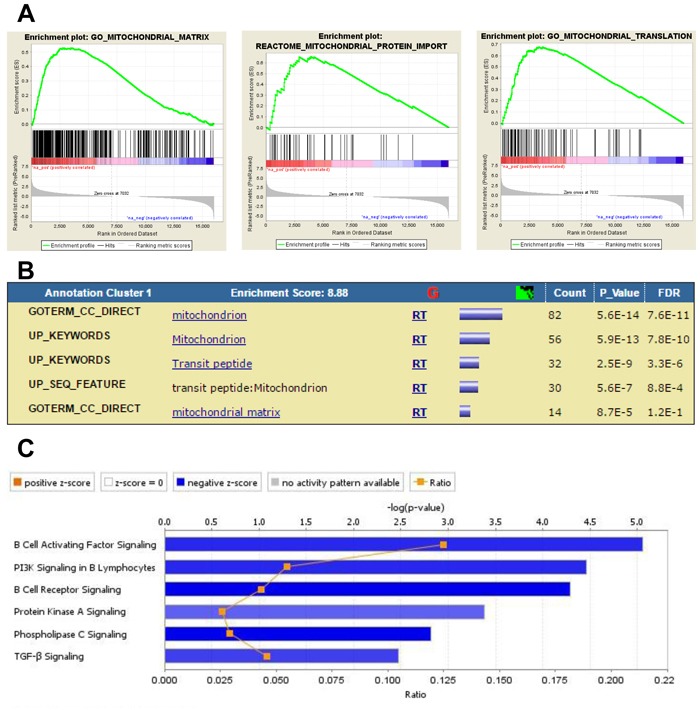
Analysis of DEG in BM-MSC from mice challenged with MLL/ENL+FLT3-ITD *Trp53*^-/-^ leukemia **A.**, Enrichment plots showing association of up- and down-regulated genes in this database with mitochondria functions. **B.**, DAVID Functional Annotation Clustering report displaying grouped annotations with the higher enrichment scores for this dataset. The p-values associated with each annotation terms inside each clusters are exactly the same meaning/values as p-values (Fisher Exact/EASE Score) shown in the regular chart report for the same terms. FDR: False Discovery Rate. **C.**, IPA Canonical Pathways analysis displaying the most significant pathways associated with down-regulated genes induced by MLL/ENL+FLT3-ITD *Trp53*^-/-^ leukemia in BM-MSC. The Stacked Bar Chart displays the down-regulated genes molecules in each Canonical Pathway with regards to their z-score. The color blue reflects a negative direction of change for the function. Z-scores ≤ -2 indicate that the function has a statistically significant decrease.

In order to determine the impact of p53 expression in leukemia cells on gene expression in BM-MSC, we compared the ways in which GEP results for BM-MSC exposed to the *Trp53* wt and *Trp53*^-/-^ forms of MLL/ENL+FLT3-ITD, as compared to control BM-MSC, differed from each other. Solely for BM-MSC exposed to MLL/ENL+FLT3-ITD null for *Trp53*, GSEA found positive enrichment for gene sets related to the activation of MYC and for gene sets associated with mitochondria functions like respiration, mitochondrial matrix, mitochondrial complex formation and mitochondrial protein import (Figure 5A and Table 2). Clustering by DAVID functional annotation confirmed these findings (Figure 5B). Other gene sets positively enriched included ones associated with ribosomal subunit assembly and biogenesis, RNA methylation, RNA translation and metabolic process as well as RNA methyltransferase activity and other RNA metabolic functions. IPA of the 866 DEGs uniquely up-regulated in BM-MSC from the MLL/ENL+FLT3-ITD *Trp53*^-/-^ condition implicated the activation of transcriptional regulators like REL (C-Rel, an NF-κB subunit) and NRF2 (activation Z-score ≥3.2, opposite to MLL-ENL condition), as well as cellular functions including hydrolysis of carbohydrates and metabolism of fatty acids and nucleic acids. Similar processes were not found for BM-MSC exposed to the isogenic leukemia cells expressing p53 wt. IPA of the 827 DEGs uniquely down-regulated in BM-MSC exposed to MLL/ENL+FLT3-ITD null for *Trp53* indicated inhibition of PI3K, PLC, TGF-β and B-cell receptor signaling, opposite to BM-MSC exposed to MLL-ENL (Figure 5C).

**Table 2:** Similarities and differences between DEG in BM-MSC from mice engrafted with either MLL/ENL+FLT3-ITD T*rp53*^-/-^ or MLL/ENL+FLT3-ITD *Trp53 wt*

MLL/ENL+FLT3-ITD *Trp53*^-/-^	MLL/ENL+FLT3-ITD *Trp53 wt*
Similarities: Activation of β-Catenin Genes up-regulated in HSC	Activation of β-Catenin Genes up-regulated in HSC
Differences: Hypoxia Activation of MYC Mitochondrial functions, ribosome assembly, RNA translation and RNA metabolic functions Activation of C-Rel and NRF2 Inhibition of PI3K, PLC, TGF-β	Activation of AHR Activation of TATA Collagen formation Activation of Polo-like kinase, Integrins, mTOR and NGF Inhibition of IL-8, IL-6, cytokines production, NFAT-mediated regulation of immune response, TLR-mediated signaling and Rho family of GTPases

Summary of changes in GEP observed in BM-MSC under the two experimental conditions. GSEA and IPA were used as analysis tools to determine similarities and differences presented.

**Figure 6:**
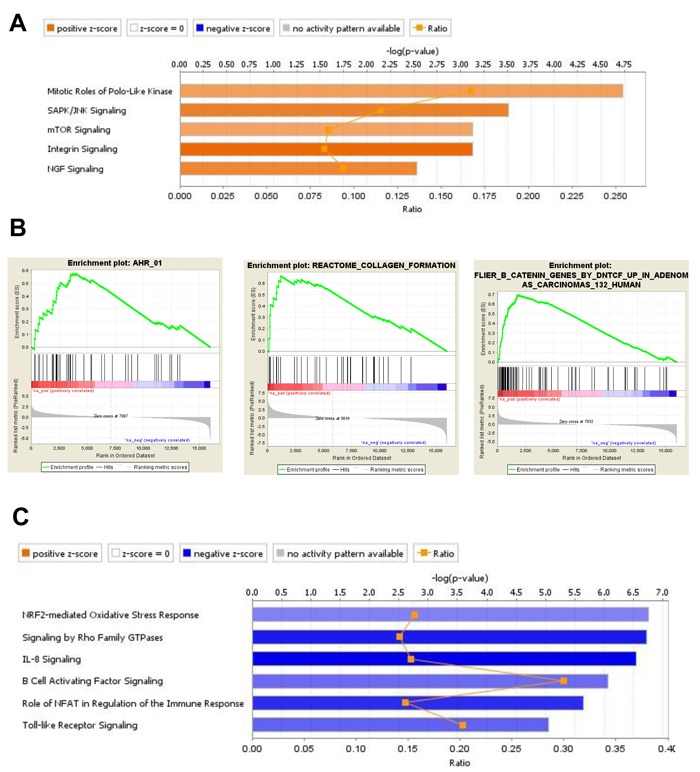
Gene expression analysis of DEG in BM-MSC from mice engrafted with MLL/ENL+FLT3-ITD *Trp53* wt leukemia **A.**, IPA displaying the most significant Canonical Pathways associated with up-regulated genes induced by MLL/ENL+FLT3-ITD *Trp53 wt* leukemia in BM-MSC. Orange equals positive direction of change for the function (Z-scores ≥ 2). **B.**, Enrichment plots exemplifying the most significant enrichment sets for up-regulated genes in this condition. **C.**, Canonical Pathways analysis (IPA) displaying the most significant pathways associated with down-regulated genes induced in BM-MSC by MLL/ENL+FLT3-ITD *Trp53 wt* leukemia (Z-scores ≤ -2).

For BM-MSC exposed to MLL/ENL+FLT3-ITD *Trp53 wt*, canonical pathway IPA of the 970 uniquely up-regulated DEGs indicated a positive role for Polo-like kinase in mitosis, activation of signaling mediated by SAPK/JNK, mTOR, integrins and NGF (Figure 6A). Interestingly, NGF (nerve growth factor) is a secreted factor that, in addition to controlling cell survival, growth, and differentiation, can regulate inflammatory responses [26] by interfering with Toll-like receptor (TLR) signaling. None of these pathways were significant when up-regulated genes from BM-MSC from the MLL/ENL+FLT3-ITD *Trp53*^-/-^ condition were analyzed. IPA for down-regulated genes showed a significant negative z-score for IL-8 and TLR -mediated signaling. Negative scores were shown as well as for the role of NFAT in the regulation of immune response and signaling by Rho family of GTPases, indicating that these signaling pathways were uniquely inhibited in BM-MSC challenged with the p53 wt-expressing leukemia sample (Figure 6C). There was a relatively small number of gene sets significantly enriched in GSEA for *Trp53 wt*, including ones associated with collagen formation (uniquely enriched), activation of aryl hydrocarbon receptor (AHR) signaling (also observed in BM-MSC exposed to AML1/ETO9a), and activation of β-catenin (Figure 6B). Interestingly, IPA results were confirmed in that there was a negative enrichment for gene sets associated with positive regulation of IL-6 and IL-8 production, indicating a decrease in the production of these cytokines and a reduced inflammatory response.

## DISCUSSION

BM-MSC are a central component of the BM microenvironment and can differentiate into at least three different cell lineages: osteoblasts, chondroblasts, and adipocytes [27–29]. They provide a nurturing niche for normal hematopoiesis by providing cell-adhesion signals, growth factors, cytokines, chemokines and other soluble factors. It has been shown that during leukemia progression, leukemia cells “hijack” the normal hematopoietic BM niche and modify it to create a tumor-permissive microenvironment [30]. Although it has been reported that BM-MSCs from patients with AML and MDS have cytogenetic abnormalities that differ from leukemic blasts and that may be associated with inferior outcomes [31], the contribution of leukemic cells to the associated transcriptional and epigenetic changes in BM-MSC is not fully understood, and, in particular, how these changes may vary with those of AML genotype.

AML cells expressing distinct genotypes display differences in response to conventional chemotherapy [32], and *Trp53* mutational status dictates sensitivity to chemotherapeutic agents [9]. However, it is likely that these differences are not entirely cell-intrinsic, i.e., that AML cells influence the surrounding BM microenvironment (including BM-MSC) in common and genotype-specific ways, and that this affects response to chemotherapy. We found that AML cells modify the transcriptional profile of BM-MSC, and do so in ways that differ with the AML genotype and/or *Trp53* status. Because we used defined human AML-associated oncogenes, isogenic WT or null *Trp53* status for one of these oncogenes, and adoptive transfer to syngeneic C57BL/6 mice, the differences in BM-MSC transcriptomes are attributable to the differences in AML genotypes. In the case of BM-MSC collected from AML1/ETO9a leukemia-bearing mice, up-regulated genes were related to cancer progression and a decrease of apoptosis. Analysis of these DEGs indicated changes in the BM-MSC associated with increased hypoxia, reduced inflammatory response and adipogenesis. Interestingly, adipogenesis was also a signature present in BM-MSC exposed to MLL/ENL and MLL/ENL+FLT3-ITD *Trp53*^-/-^ leukemia but not in BM-MSC from MLL/ENL+FLT3-ITD *Trp53* *wt* leukemia-bearing mice. In line with these data, abundant evidence shows that many tumors grow in the vicinity of adipocytes or metastasize to adipocyte-rich environments which act as an important reservoir of triglycerides [33]. The hydrolysis of these triglycerides produces free fatty acids used as an energy source by adjacent cancer cells [34, 35]. The oxidation of these fatty acids becomes a crucial mechanism on which cancer cells depend to survive in nutrient- and oxygen-depleted environmental conditions [36] such as the hypoxic BM-microenvironment, suggesting potential for therapeutic strategies aimed to exploit the lipid-related metabolic dependence.

For BM-MSC collected from mice challenged with MLL/ENL leukemia, hypoxia, PI3K and NF-κB activation were also a prevalent signature. Remarkably, GSEA indicated activation of β-catenin and inhibition of MYC. In addition, genes associated with stromal stimulation and ECM proteins were enriched in this set. Up-regulation of genes coding for ECM proteins was also a feature in BM-MSC exposed to MLL/ENL+FLT3-ITD leukemia (both *Trp53 wt* and *Trp53* ^-/-^), with *BMP1, SPON2,* and *TGM2* being the most prominent. *SPON2* and its translational product, Spondin-2, have been found to be up-regulated in many types of cancer [37–39], and its expression is a prognostic factor in prostate and colorectal cancer [40, 41]. In breast cancer, expression of TGM2 in tumor stroma is an independent risk factor for breast cancer patients at high risk of recurrence [42]. In AML, TGM2 was found up-regulated particularly in relapsed patients and correlated with increased expression of numerous adhesion proteins and many apoptosis regulating proteins [43]. Moreover, TGM2 overexpression activates NF-κB and HIF1A leading to chemoresistance [44, 45].

Within the MLL/ENL+FLT3-ITD groups, the activation of β-catenin was a common feature and was also present in the MLL/ENL group. Interestingly, β-catenin is known to regulate proliferation and differentiation of BM-MSC [46] and the expression of an activated form of β-catenin in mouse osteoblasts resulted in the development of MDS and secondary AML in mice [47]. Remarkably, the presence or absence of p53 in these leukemia cells had a different impact in the GEP of BM-MSC. Genes associated with mitochondrial functions, RNA translation and RNA metabolic functions were significantly enriched in BM-MSC from mice challenged with MLL/ENL+FLT3-ITD *Trp53*^-/-^ but not in BM-MSC from the MLL/ENL+FLT3-ITD *Trp53* *wt* condition. The presence of p53 in this condition was associated with transcriptome changes in BM-MSC characterized by a decrease in IL-8, IL-6, NFAT and TLR-mediated signaling consistent with a reduced inflammatory response and an immunosuppressive microenvironment. A similar trend was observed in BM-MSC exposed to AML1/ETO9a leukemia. Other differences include activation of mTOR and NGF in the MLL/ENL+FLT3-ITD *Trp53* *wt* condition and inhibition of PI3K, PLC and TGF-β signaling in the *Trp53*^-/-^ counterpart (Table 2). Activation of NRF2 in BM-MSC exposed MLL/ENL+FLT3-ITD *Trp53*^-/-^ was a key feature of this condition and opposite to BM-MSC challenged with MLL/ENL leukemia suggesting that this signature could be driven by FLT3-ITD but counteracted by p53 as it was not significant in the MLL/ENL+FLT3-ITD *Trp53* *wt* condition. Due to the role of in NRF2 in mediating oxidative stress response and the production of reactive oxygen species (ROS), both considered mediators of drug resistance and metastasis in cancer [48, 49], the use of antioxidant supplements, enzymes, and inhibitors for ROS-generating NADPH oxidases (NOX) could be a logical therapeutic intervention for AML patients with these particular genetic abnormalities.

Despite differences in genotype and *Trp53* status between the four types of syngeneic primary AML, BM-MSC isolated from leukemia-bearing mice shared certain transcriptional changes when compared to BM-MSC isolated from healthy control mice. Notably, all 4 leukemias induced the up-regulation of *CXCL12* in BM-MSC. SDF-1α, the protein product of *CXCL12,* controls the trafficking of hematopoietic stem cells (HSC) and leukemia stem cells (LSC) from and to the BM cavity through the binding to CXCR4. High SDF-1α expression defines a subset of perivascular BM-MSCs called CXCL12-abundant reticular (CAR) cells, which are important for controlling HSC proliferation [50, 51] and maintenance [52]. Moreover, activation of the SDF-1α/CXCR4 axis is essential for the migration and survival of both normal and leukemic hematopoietic cells *in vivo* and *in vitro* [53, 54]. Overexpression of CXCR4 in AML correlates with poor clinical outcome [55, 56] and hence, inhibition or blockade of the SDF-1α/CXCR4 axis has become an attractive therapeutic approach for AML [57–59] showing efficacy in preclinical studies [60] and in an ongoing clinical trial with BL-8040 [61]. Our group demonstrated that the activation of this axis upregulates the transcription regulator Yin Yang 1 (YY1), which represses transcription of the miRNA let-7a, leading to enhanced expression of MYC and the anti-apoptotic protein BCL-XL in AML cells [62].

Other genes up-regulated by all leukemia genotypes in BM-MSC were *CTGF*, *Igfbp5* and *MGP*. Interestingly, both CTGF and Igfbp5 have been independently found to inhibit osteoblast maturation and differentiation [63, 64] while *MGP* is an ECM protein that has been shown to inhibit mineralization and apoptosis of chondrocytes [65]. These findings suggest that interactions between leukemia and the BM stroma induce changes towards an undifferentiated state of the tumor microenvironment that could contribute to disease progression and chemotherapy resistance [66]. Furthermore, our findings indicate up-regulation of genes related to complement (*C4A, C4B,* and *SERPING1*) in BM-MSC exposed to leukemia *in vivo*. Consistent with our data, it has been recently shown that cancer cells can activate complement and such activation can promote an immunosuppressive microenvironment, induce angiogenesis and activate cancer-related signaling pathways [reviewed in [67]]. In this chronic inflammation-like state that resembles “a wound that does not heal” [14], stromal cells accumulate certain types of cytokines, chemokines, growth factors, matrix remodeling proteases, and reactive oxygen species critically compromising tissue homeostasis and creating a tumor-supportive microenvironment [68].

In summary, our results support the hypothesis that AML regulates the transcriptomes of BM-MSC, in both shared and genotype-specific ways. A caveat of our experimental design is that our healthy control mice were not irradiated and hence some of the gene expression changes in BM-MSC from leukemia-bearing mice could be attributed to the effects of radiation [69, 70]. Nonetheless, the distinct gene expression signatures obtained in BM-MSC challenged with different leukemia genotypes suggest that most of these changes were specific and leukemia-driven. Moreover, these changes are likely to happen exclusively *in vivo* when all the components of the microenvironment are present; conditions that are almost impossible to recreate *in vitro*. Accordingly, our *in vitro* 2-D co-culture experiments failed to validate most of the gene expression changes detected *in vivo,* except for *ICAM1* and *ITGB7* which were found to be up-regulated *in vivo* (Supplementary Table 1) and *in vitro* (Supplementary Figure 4). Although the results warrant further analysis and validation, the data presented here demonstrate for the first time that differences in the genotype and p53 status of leukemia cells can have a distinct impact on how the BM microenvironment responds to AML, but also indicate that many changes in the BM stroma are common to different leukemia genotypes. Consistent with our observations, reverse phase protein array analysis (RPPA) carried out by our group [71] comparing more than 150 proteins (including phosphorylated state when available) indicated that the expression of a distinct group of proteins including HIF1A, FOXO3, BCL2, TRP53, MAPK and STAT5 (phospho-Tyr694) was elevated in AML-derived BM-MSC (*n* = 106) compared to normal donor-derived BM-MSC (*n* = 71). Interestingly, we identified in this dataset 5 AML patients with 11q abnormalities (MLL rearrangements) which clustered in close proximity. At P value of 0.05, only 4 proteins were differentially expressed in this group and only CDK4 was consistently up-regulated in all 5 samples (Supplementary Figure 3). In agreement with these data, *CDK4* mRNA was up-regulated in BM-MSC from mice engrafted with MLL/ENL+FLT3-ITD *Trp53 wt* and MLL/ENL+FLT3-ITD *Trp53* ^-/-^ but not in BM-MSC from other leukemia-bearing mice. Given that AML development, progression, and therapy resistance involves the evolution of tumor subclones, our findings suggest that the relative effects of different subclones on BM-MSC may contribute to their selection. Understanding these effects may facilitate the development of therapeutic strategies to render the BM microenvironment inhospitable to leukemia cells but supportive of normal hematopoiesis.

## MATERIALS AND METHODS

### Mice

C57BL/6 mice were obtained from The Jackson Laboratory. All protocols concerning animal use were approved by the Institutional Animal Care and Use Committee at The University of Texas MD Anderson Cancer Center and conducted in accordance with the NIH Guide for the Care and Use of Laboratory Animals.

### Engraftment of primary leukemia and mouse BM-MSC isolation

Syngeneic primary leukemia cells were kindly provided by Scott Lowe and they were generated as previously described [9]. In brief, these primary leukemia cells were generated by introducing the desired human oncogenic translocations *via* retroviral transduction into fetal liver cells (FLC) derived from embryonic day 13.5-15 (E13.5-E15) embryos. FLCs were obtained from either Trp53 knockout (*Trp53* ^-/-^) C57BL/6 mice (AML1/ETO9a, MLL/ENL, MLL/ENL+FLT3-ITD) or *Trp53 wt* C57BL/6 mice (MLL/ENL+FLT3-ITD *Trp53 wt*). Retroviral constructs harboring the mentioned translocations were engineered to co-express GFP. FLCs were co-transduced with retrovirus encoding Firefly Luciferase and Nras^G12D^ separated by an internal ribosomal entry site (IRES). Co-transduced FLCs, which are highly enriched for hematopoietic stem and progenitor cells [72], were used to reconstitute the hematopoietic compartment of lethally irradiated syngeneic recipient mice. *In vivo* expanded genetically-modified leukemia cells were transplanted into 10 mice (6- to 8-wk-old) by tail-vein injection of 1 × 10^6^ cells/per recipient. PBS was injected in the control group. Mice were sub-lethally irradiated prior to the injections to favor a uniform disease onset in recipient animals. Leukemia engraftment and burden was subsequently monitored by noninvasive imaging of isoflurane-anesthetized mice injected intraperitoneally with D-Luciferin using an *in vivo* imaging system (IVIS system; Xenogen/Caliper Life Sciences)

### Mouse BM-MSC isolation and FACS

Transplanted leukemia was allowed to progress for 2 (MLL/ENL and MLL/ENL+FLT3-ITD), 3 (AML1/ETO9a) or 4 weeks (MLL/ENL+FLT3-ITD *Trp53 wt)* according to disease burden. At this time, mice were sacrificed and BM-MSC were obtained by flushing mice femurs with saline. MSC from different leukemia-bearing mice were isolated by FACS using a combination of the following cell surface markers: Ter119-, CD31-, CD45-, CD105+, Sca1+, CD106+ and PDGF-Rα+ (CD140) [11, 12] (Supplementary Figure 1).

### *In vitro* co-culture of mouse BM-MSC and syngeneic primary leukemia cells

C57BL/6-derived BM-MSC were co-cultured *in vitro* with each of the primary leukemia cells harboring the different AML genotypes that were used in the *in vivo* study. Monocultures of BM-MSC were also incubated under the same conditions and used as controls. BM-MSC were cultured alone (monoculture) or co-cultured for 48 h with the corresponding leukemia cells at an initial ratio of 1:4 (BM-MSC to leukemia cells). After culture, CD45- CD105+ BM-MSC were isolated by FACS. Total RNA was extracted from cells using Trizol (Invitrogen, ThermoFisher, Carlsbad, CA) as directed by the manufacturer and reverse transcribed as previously described [73].

### Real-Time PCR

Messenger RNA levels were quantified by TaqMan gene expression assays run in duplicate using an ABI Model 7900HT Fast Real-Time PCR System as directed by the manufacturer (Applied Biosystems, ThermoFisher, Foster City, CA), and data were analyzed by the 2-ΔΔCt using RQ Manager version 1.2.1 (Applied Biosystems).

### Gene expression profiling (GEP)

Total RNA was isolated from sorted and pooled BM-MSC using the RNAqueous-Micro kit (Ambion). After confirmation of RNA quality using a Bioanalyzer 2100 instrument (Agilent), 100 ng or less of total RNA was amplified and biotin-labeled through a two-round Eberwine procedure using MessageAmp II and Illumina TotalPrep RNA Amplification kits (Ambion), and hybridized to Illumina Ref-6 version 2 mouse whole-genome arrays. Processing of bead-level data was by methods previously described [74]. Complete dataset can be found at GEO (Submission GSE97194).

Overlap between AML genotypes in differentially-expressed genes was displayed using InteractiVenn (http://www.interactivenn.net/index.html) [75].

## SUPPLEMENTARY MATERIALS FIGURES AND TABLES














