# Supplementary material for: Tumor Trp53 status and genotype affect the bone marrow microenvironment in acute myeloid leukemia
Source: Oncotarget. 2017 Jul 6;8(48):83354–69. doi: 10.18632/oncotarget.19042 (PMC5663521; doi:10.18632/oncotarget.19042)
Supplement: Supplementary file 1 [file oncotarget-08-83354-s001.pdf]

Tumor *Trp53* status and genotype affect the bone marrow microenvironment in acute myeloid leukemia

Supplementary Material

Healthy Control Mice

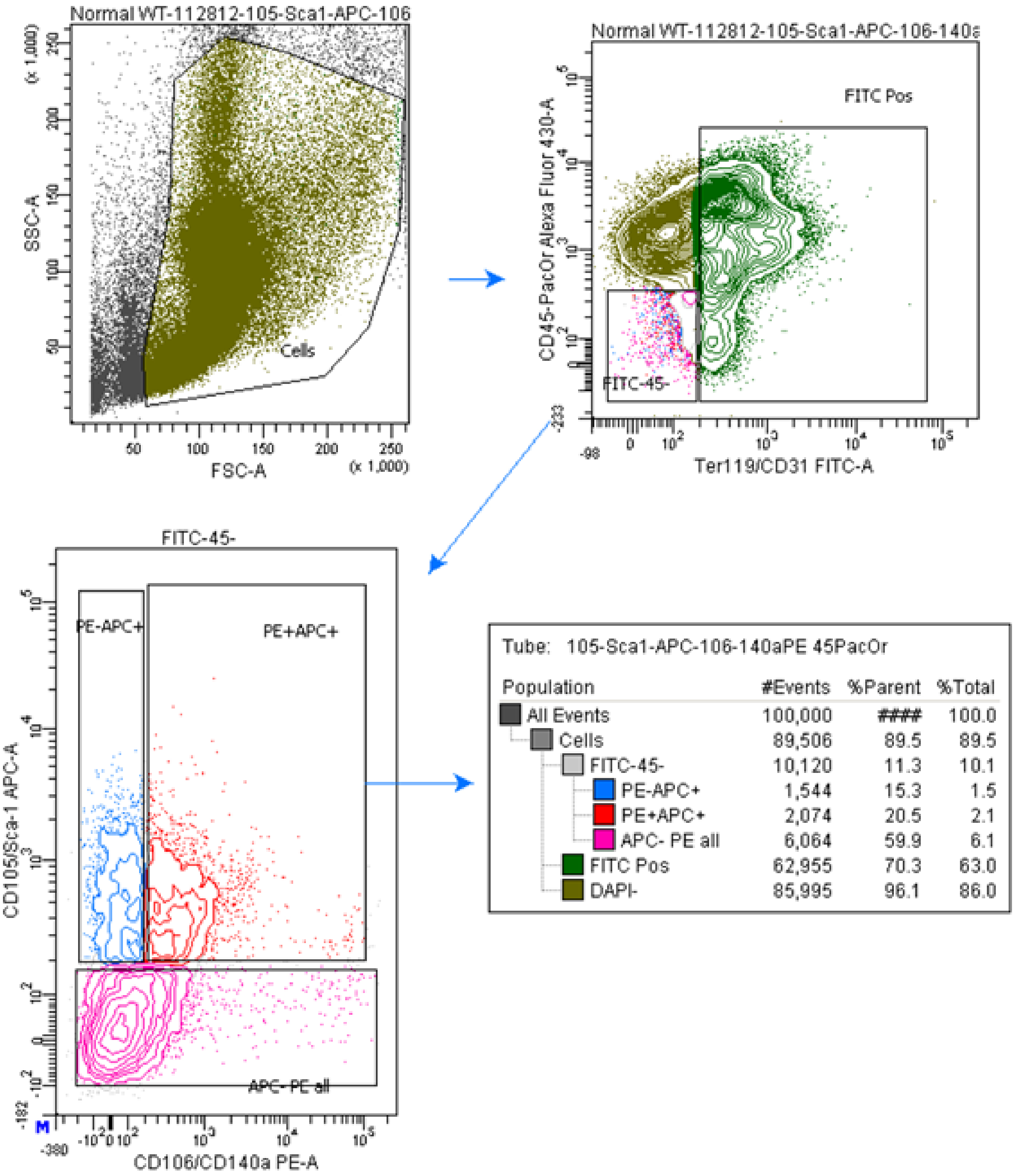

Population	#Events	%Parent	%Total
All Events	100,000	####	100.0
Cells	89,506	89.5	89.5
FITC-45-	10,120	11.3	10.1
PE-APC+	1,544	15.3	1.5
PE+APC+	2,074	20.5	2.1
APC- PE all	6,064	59.9	6.1
FITC Pos	62,955	70.3	63.0
DAPI-	85,995	96.1	86.0

AML1/ETO9a-engrafted Mice

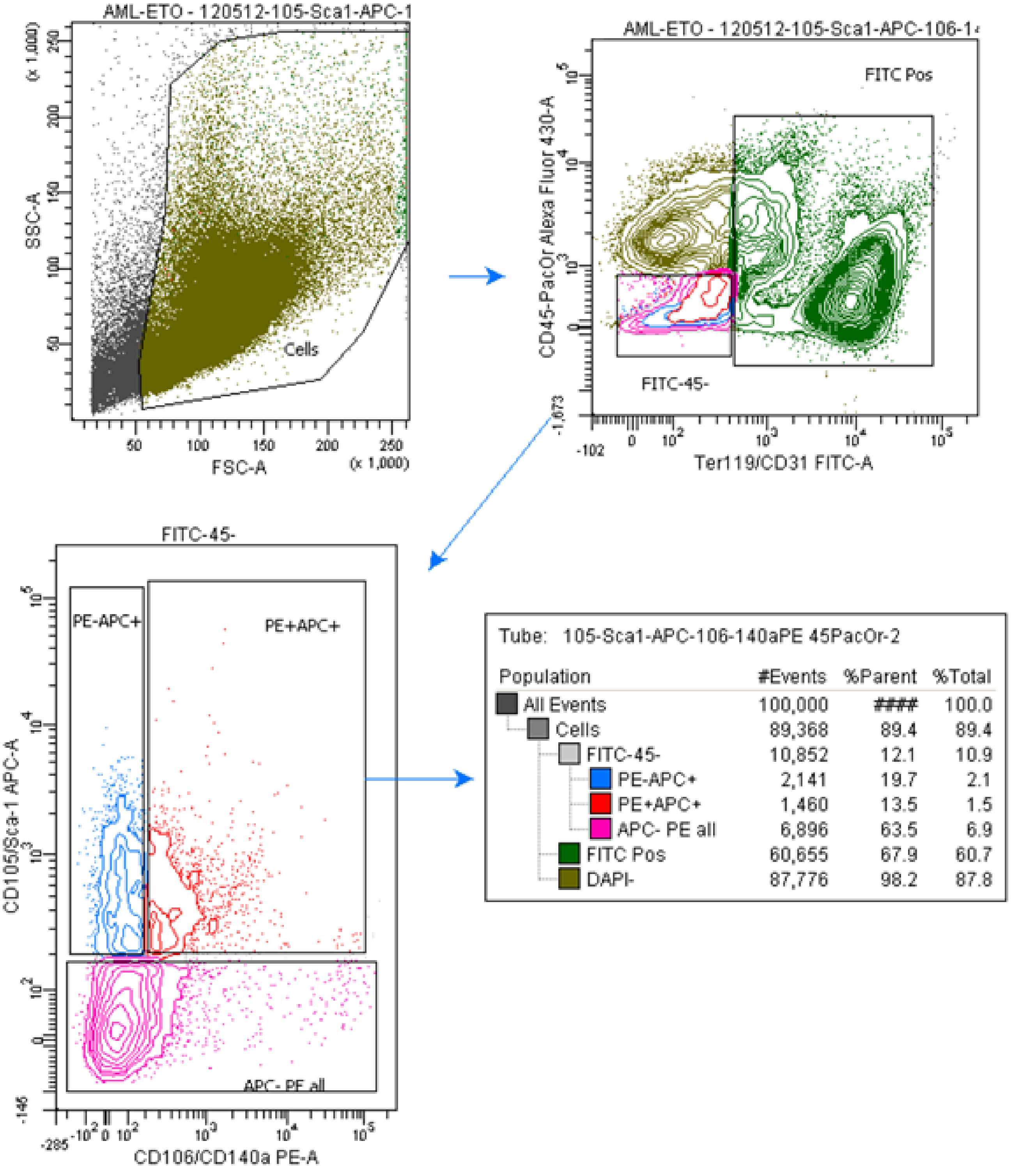

Tube: 105-Sca1-APC-106-140aPE 45PacOr-2

Population	#Events	%Parent	%Total
All Events	100,000	####	100.0
Cells	89,368	89.4	89.4
FITC-45-	10,852	12.1	10.9
PE-APC+	2,141	19.7	2.1
PE+APC+	1,460	13.5	1.5
APC- PE all	6,896	63.5	6.9
FITC Pos	60,655	67.9	60.7
DAPI-	87,776	98.2	87.8

MLL/ENL-engrafted Mice

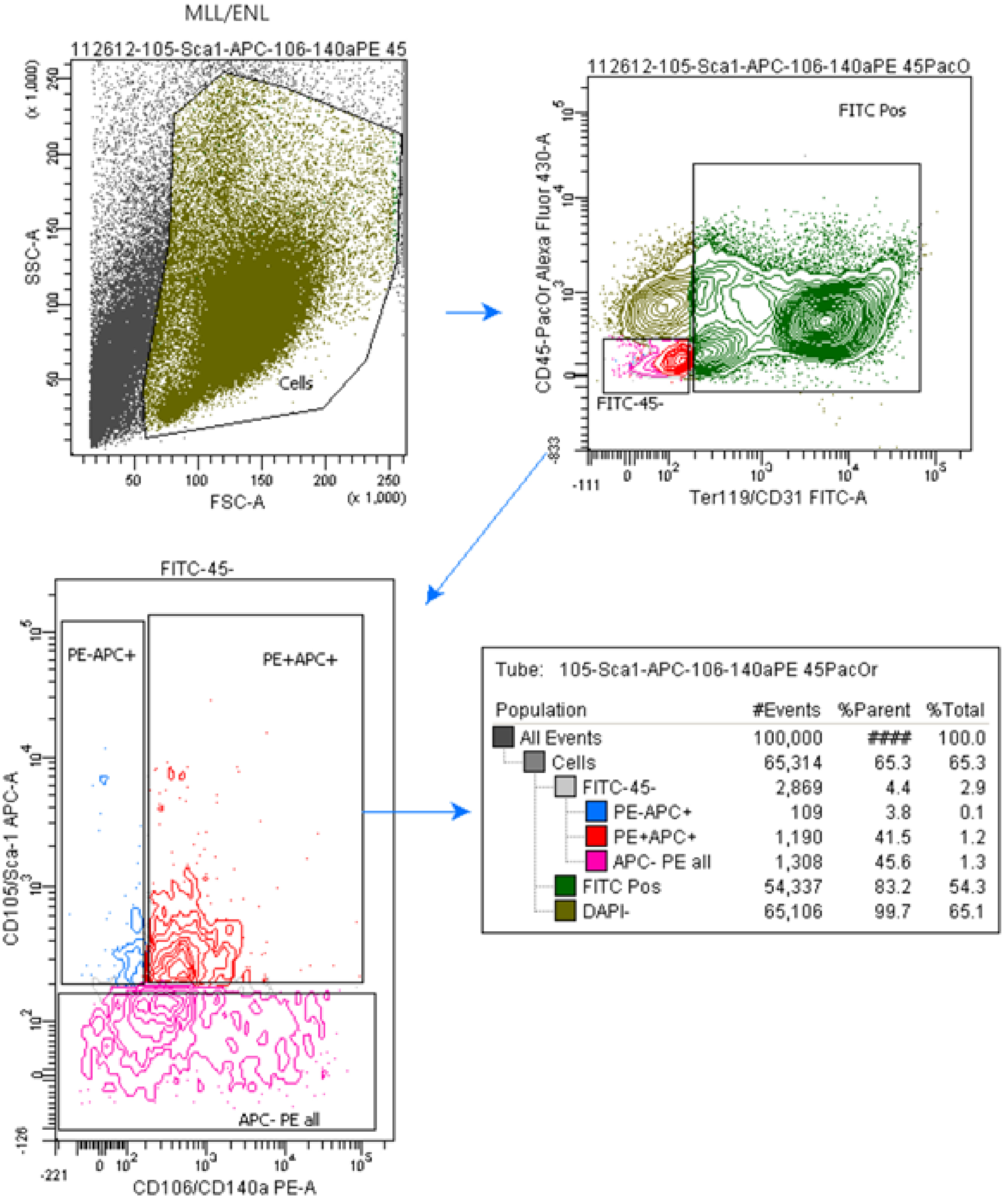

Tube: 105-Sca1-APC-106-140aPE 45PacOr

Population	#Events	%Parent	%Total
All Events	100,000	####	100.0
Cells	65,314	65.3	65.3
FITC-45-	2,869	4.4	2.9
PE-APC+	109	3.8	0.1
PE+APC+	1,190	41.5	1.2
APC- PE all	1,308	45.6	1.3
FITC Pos	54,337	83.2	54.3
DAPI-	65,106	99.7	65.1

MLL/ENL-FLT3-ITD *Trp53 -/-* engrafted Mice

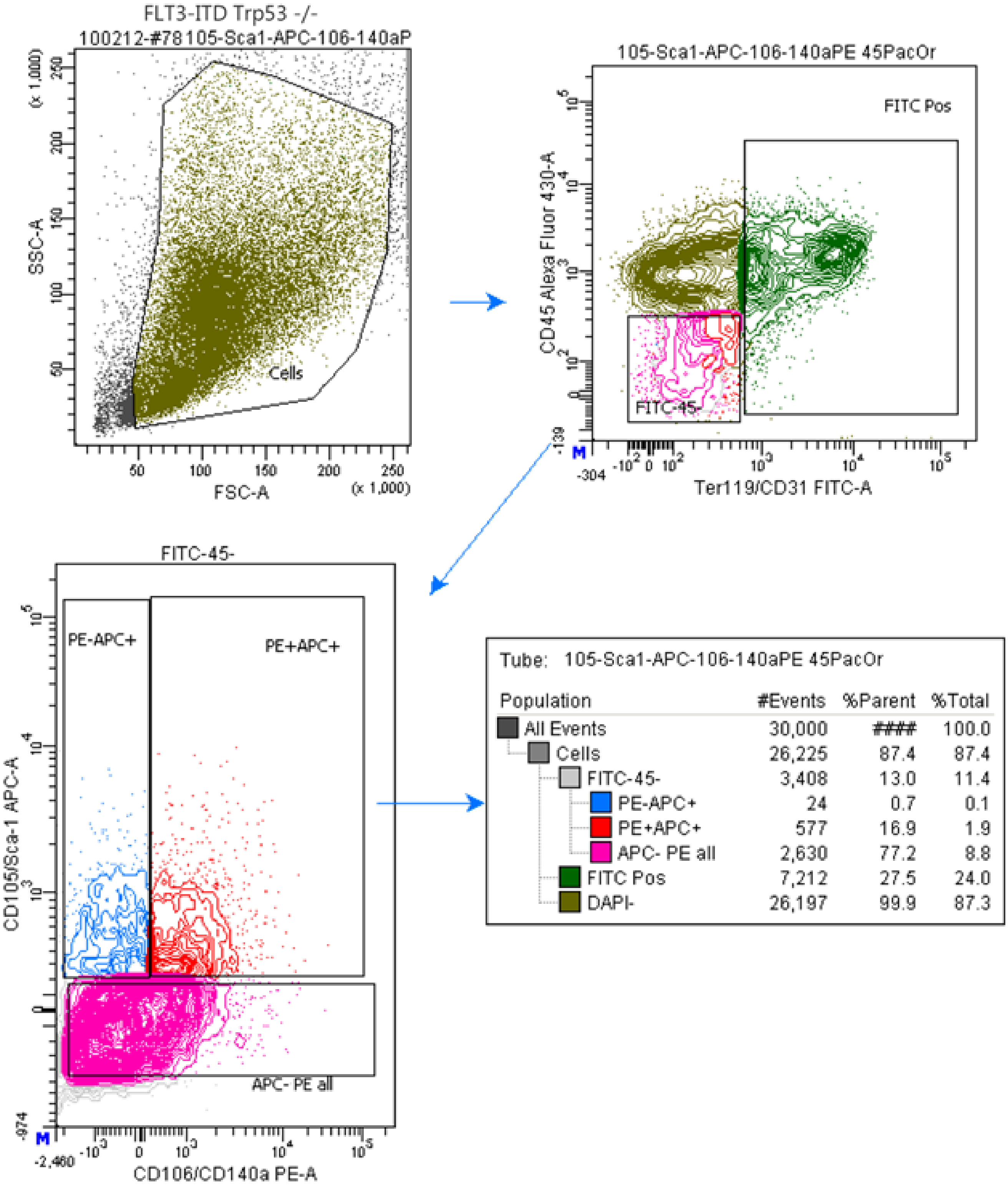

Tube: 105-Sca1-APC-106-140aPE 45PacOr

Population	#Events	%Parent	%Total
All Events	30,000	####	100.0
Cells	26,225	87.4	87.4
FITC-45-	3,408	13.0	11.4
PE-APC+	24	0.7	0.1
PE+APC+	577	16.9	1.9
APC- PE all	2,630	77.2	8.8
FITC Pos	7,212	27.5	24.0
DAPI-	26,197	99.9	87.3

MLL/ENL-FLT3-ITD *Trp53 wt* engrafted Mice

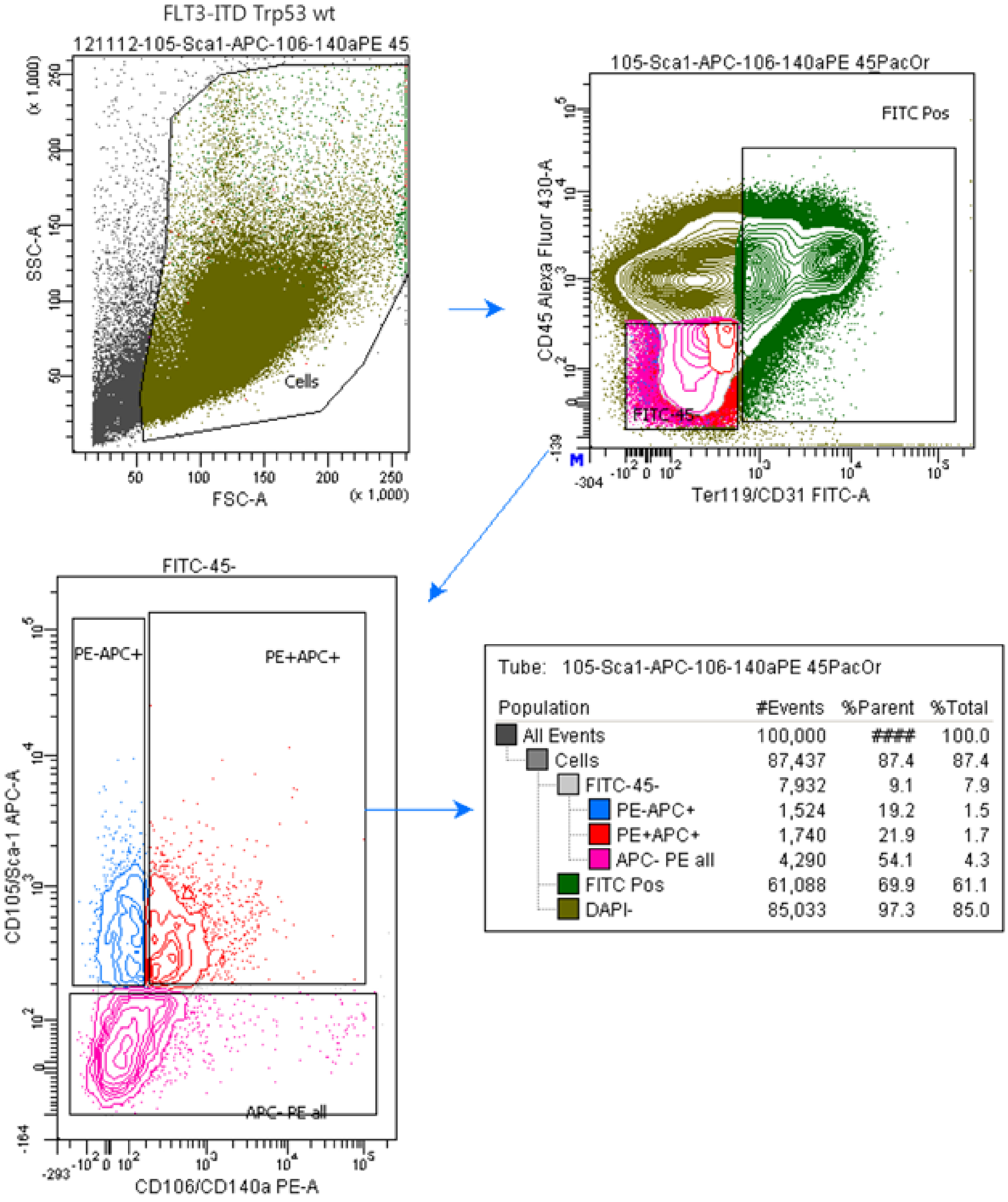

Tube: 105-Sca1-APC-106-140aPE 45PacOr

Population	#Events	%Parent	%Total
All Events	100,000	####	100.0
Cells	87,437	87.4	87.4
FITC-45-	7,932	9.1	7.9
PE-APC+	1,524	19.2	1.5
PE+APC+	1,740	21.9	1.7
APC- PE all	4,290	54.1	4.3
FITC Pos	61,088	69.9	61.1
DAPI-	85,033	97.3	85.0

Supplemental Figure 1: **Representative FACS plots for BM-MSC isolation.** 2 to 4 weeks after confirmation of leukemia engraftment, multiple mice from each condition were sacrificed and BM-MSC were isolated and sorted by FACS based on cell surface markers: GFP-, Ter119-(FITC), CD31-(FITC), CD45-(Alexa-Fluor 430), CD105+ (APC), Sca1+ (APC), CD106+ (PE), PDGF-Rα+ (CD140-PE).

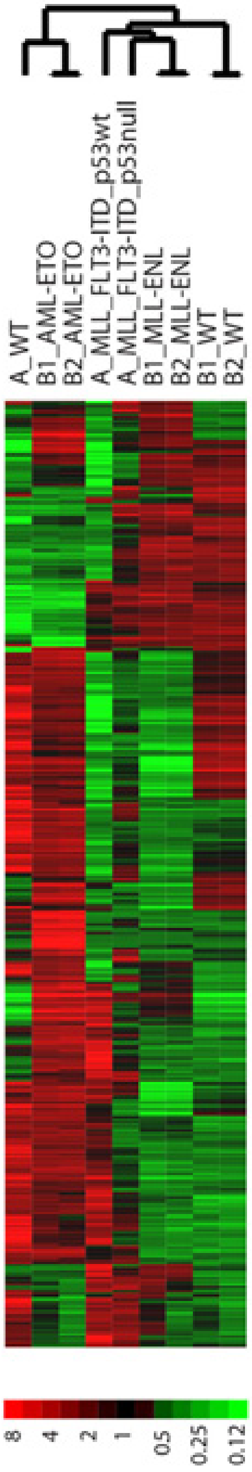

Supplemental Figure 2: **Heat map of expression of highly variant genes in control or AML-exposed BM-MSCs**. GEP data were generated from purified BM-MSCs in two experiments (A and B), each of which included control mice without AML (WT) and mice bearing AML of specific genotypes. Duplicate pools in experiment B are indicated by number. Log2-transformed data were filtered for gene probes for which at least 6 of the 9 samples had a mean-centered absolute log2 value of at least 1. The samples and 708 probes found were hierarchically-clustered, and their expression values are displayed in the heat map according to fold-change from the mean (shown in the color bar). The high similarity and adjacent clustering of duplicate pools indicate data quality, and support the averaging of data from duplicates.

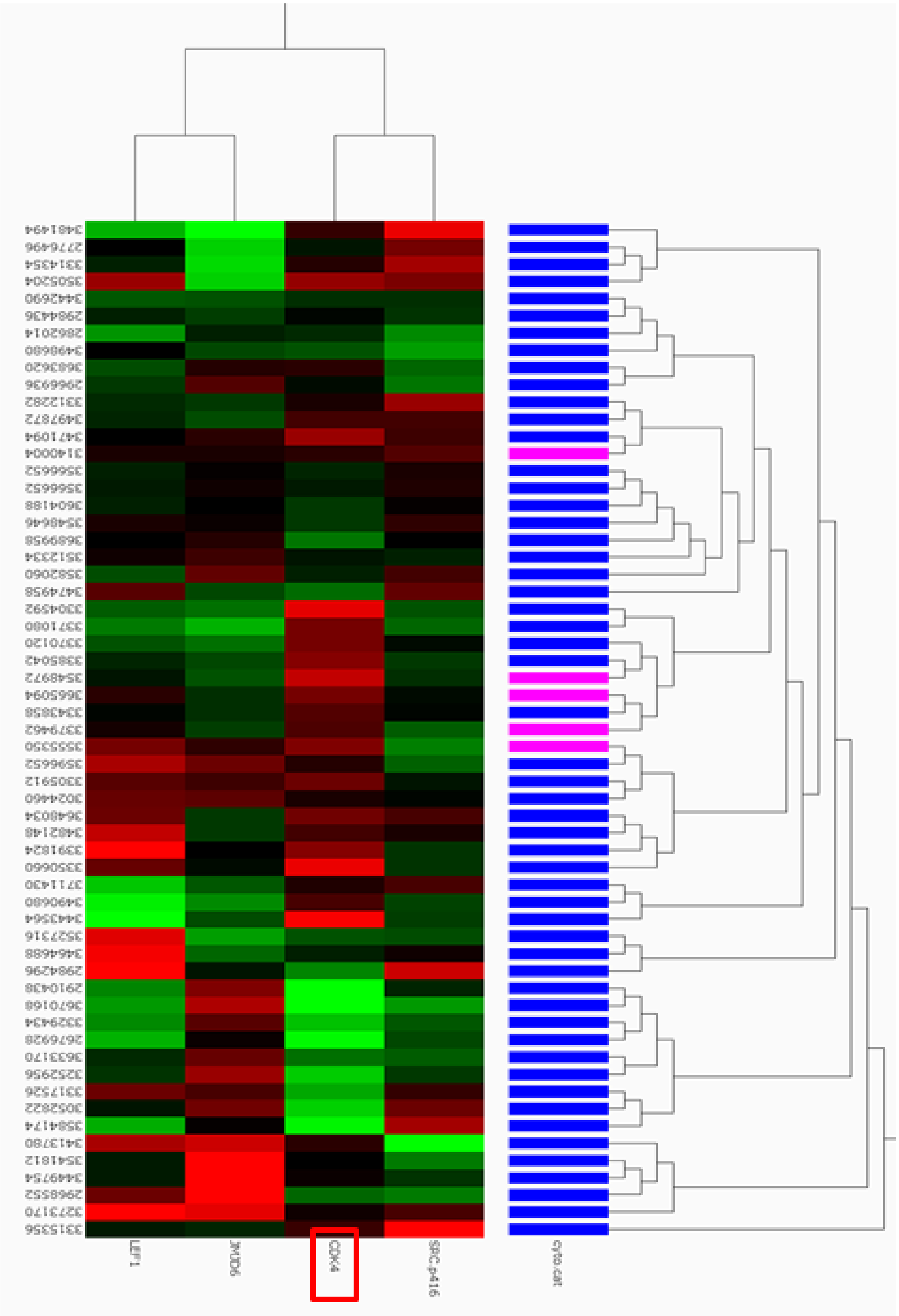

Supplemental Figure 3: **RPPA data from AML-derived BM-MSC**. Unbiased hierarchical clustering was performed in a cohort of AML-derived BM-MSC (n=106) using established algorithms (ref 1 & 2 below), with mean set to 0 and variance set to 1, looking for proteins with differential expression between the Diploid cases (blue) and those with 11q abnormalities (pink), at the p = 0.05 level. CDK4 (highlighted) was found up-regulated in all 5 samples with 11q abnormalities.

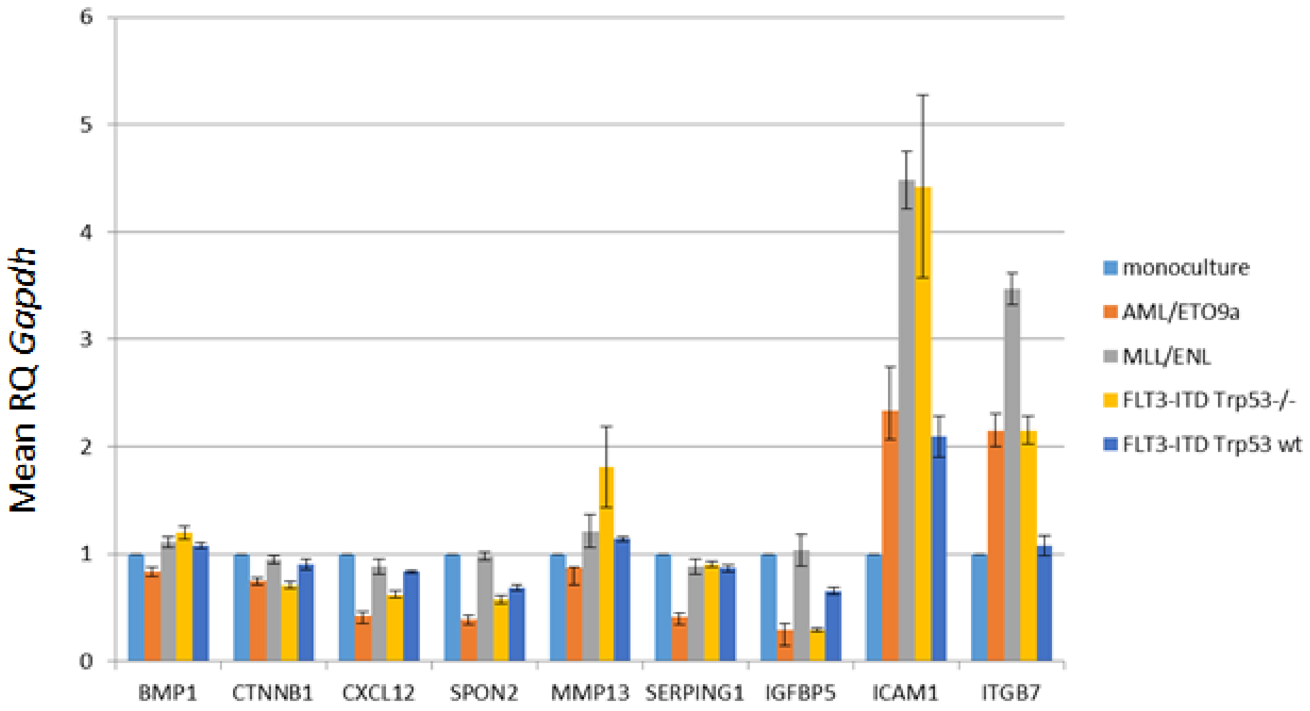

Supplemental Figure 4: **Transcriptional changes induced in mouse BM-MSC by *in vitro* co-culture with syngeneic leukemia cells**. Real-Time PCR was performed using cDNA obtained from C57BL/6-derived BM-MSC that were co-cultured *in vitro* with the indicated primary leukemia cells for 48hs and isolated by FACS as indicted in Methods. Gene expression was to normalized to GAPDH expression and presented as the mean RQ value (±SEM) of three independent experiments relative to the expression in BM-MSC cultured alone (mono-culture).

For Supplementary Tables 1,2,3,4,5,6 see in Supplementary Files

Supplementary Table 1: Subtracted data showing differentially expressed genes in BM-MSC isolated from mice challenged with AML cells with different genotypes. Gene probes with a fold-change from control (up or down) of at least 2 in at least one genotype were identified and were evaluated with respect to similarity and differences between AML genotypes in their effect on gene expression by BM-MSC. Up-regulated genes values (yellow to red) and down-regulated gene values (light green to dark green) are shown.

Supplementary Table 2: DAVID Functional Annotation Chart report of commonly up- and down-regulated genes induced in BM-MSC by all 4 genotypes. Table shows the most significant enrichment sets (Terms) for the genes in the analysis list based on comparisons with the original databases (Categories).

Supplementary Table 3: IPA molecular and cellular function analysis for genes up-regulated in BM-MSC exposed to AML1/ETO9a leukemia. The table displays the high-level functional categories with the most significant functions at the top of the list. The categories with the most significant p-values and significant z-scores are displayed. Z-scores ≥ 2 indicate that the function is statistically significantly increased (in red) and Z-scores ≤ -2 indicate decrease (green). The p-value measures the likelihood that the association between a set of genes in the query and a related function is due to random association. Genes from the analysis list that are represented in each category are listed (molecules).

Supplementary Table 4: Gene sets implicated by GSEA for the various AML genotypes. GEP data for BM-MSC from each AML genotype were separately compared by GSEA to control BM-MSC, after conversion to human homologs, using Molecular Signatures Database categories (C2, C3, C5, C6, and H) and our custom gene set collection from the literature. For each genotype, significantly enriched gene sets with an FDR q value <= 0.05 were selected, and compiled for comparison by overlap between genotypes, separately for positive and negative enrichment. Highlighted numbers shown are the NES for the significantly-enriched gene sets, color-coded from green (least significant) to red (most).

Supplementary Table 5: IPA Canonical Pathway analysis for genes up-regulated in BM-MSC exposed to MLL/ENL leukemia. Most significant pathways for up-regulated genes from this dataset (Column A) and their represented molecules (Column F) are listed and organized by their z-score value. The colors reflect the direction (orange=activation and green=inhibition) of change for the pathway with Z-scores ≥ 2 indicating that the pathway has a statistically significant increase and Z-scores ≥ -2 indicating significant decrease. The significance values (-log of p-value, column B) for the canonical pathways is calculated by Fisher's exact test right-tailed.

Supplementary Table 6: IPA Upstream Regulator analysis for genes up-regulated in BM-MSC exposed to MLL/ENL leukemia. Predicted upstream regulators of DEG in this dataset. The predicted activation state (based on z-score) reflects the direction (activation or inhibition) of the transcriptional regulator based on z-scores where values greater than 2 (activation) or smaller than -2 (inhibition) can be considered significant.
